# Intracellular cytokine detection based on flow cytometry in hemocytes from *Galleria mellonella* larvae: A new protocol

**DOI:** 10.1371/journal.pone.0274120

**Published:** 2022-09-29

**Authors:** Anna Katarzyna Wrońska, Agata Kaczmarek, Justyna Sobich, Sylwia Grzelak, Mieczysława Irena Boguś

**Affiliations:** Witold Stefański Institute of Parasitology, Polish Academy of Sciences, Warsaw, Poland; East China Normal University School of Life Sciences, CHINA

## Abstract

Invertebrates are becoming increasingly popular models for research on the immune system. The innate immunity possessed by insects shows both structural and functional similarity to the resistance displayed by mammals, and many processes occurring in insect hemocytes are similar to those that occur in mammals. However, the use of insects as research models requires the development of methods for working with hemocytes. The aim of this study was to develop a protocol for intracellular cytokine detection in *Galleria mellonella* larvae hemocytes based on flow cytometry. It describes the anticoagulant composition of the buffer, the optimal conditions for hemocyte permeabilization and fixation, as well as the conditions of cell centrifugation to prevent cell disintegration. A key element is the selection of staining conditions, especially the length of the incubation time with the primary antibody, which turned out to be much longer than recommended for mammalian cells. The development of these individual steps allowed for the creation of a reproducible protocol for cytokine detection using flow cytometry in wax moth hemocytes. This will certainly facilitate the development of further protocols allowing for wider use of insect cells in immunological research.

## Introduction

Intracellular cytokine detection by flow cytometry (ICCS) has emerged as the main technique for studying cytokine production at the single-cell level. This method has lower sensitivity than other methods, such as ELISA, ELISpot and PCR. However, ICCS enables the simultaneous detection of the specific subset of responder cells, their associated markers of differentiation (e.g. markers of memory phenotype or activation state) and the function (e.g. cytokine production, cytotoxicity-associated markers etc.); it also can be used to simultaneously identify multiple cytokines/chemokines and markers of proliferation [1]. It is now the gold standard method in mammalian immune response research, especially in the preclinical and clinical phases of vaccine evaluation, and has replaced most traditional methods for quantifying immune reactivity to specific antigens [2]. Thus, intracellular cytokine detection by flow cytometry in human cells is a relatively common procedure and many protocols, reviews and research papers have been prepared for this method. Both commercially-available ICCS kits and literature-described ICCS methods generally involve the following: (1) cell preparation, (2) immobilization of the cytokine within the cell by fixation, and (3) cell permeabilization to gain access to the intracellular cytokine, followed by (4) immunostaining with anti-cytokine antibodies [3].

There is great interest in developing immunological methods that require fewer mammal donors or use non-mammalian ones, for ethical and financial reasons. As such, the use of invertebrates, especially insects, is becoming an increasingly common alternative. One such insect model is *Galleria mellonella*, known colloquially as the wax worm/moth [4]. Many components of the immune system of *G*. *mellonella* larvae are similar to mammalian immune mechanisms: the insect cuticle acts as barrier to pathogens in a similar way as mammalian skin, and insect hemolymph can be partly compared to blood insofar that both tissues contain immunocompetent cells.

The cellular immunity system in *G*. *mellonella* is based on phagocytosis, nodulation and encapsulation reactions, and requires the presence of five types of hemocytes, each of which plays a different function in the immune response. Of these, prohemocytes are considered as multipotent progenitor cells or stem cells giving rise to the other subsets. Granulocytes and plasmatocytes constitute more than 50% of the hemocytes circulating in the hemolymph. Both are involved in the cellular immune response, and are the only hemocytes capable of adhesion. After adhesion to various surfaces, the plasmatocytes form pseudopodia, with their shape changing from circular to 10–15 μm wide and 20–30 μm long. The two remaining hemocyte types, spherulocytes and oenocytoids, are non-adherent. Spherulocytes contain numerous round and oval spheres bound to the cell membrane and are involved in the transport of cuticle components. Oenocytoids are round or oval cells with a small nucleus; they are believed to be involved in the melanization process, as they contain components of the phenoloxidase system [5].

The use of insects as research models requires the development of methods for working with hemocytes. Due to the characteristic features of hemocytes, i.e. the formation of pseudopodia or their tendency to rapid melanization, it is not possible to apply the same methods developed for mammalian leukocytes. Many studies of insect cells employ flow cytometry; the method allows a precise and accurate estimate to be made of the insect genome size, and serves as the first step in a complete genome sequencing project [6–8]. In addition, flow cytometry is used to test sperm viability in honey bee [9] and to analyze Drosophila eye discs [10]. Some studies also report the use of flow cytometry in the study of insect hemocytes: for characterizing the hemocyte subpopulation in *Bombyx mori* [11, 12], for honey bee hemocyte profiling [13], for hemocyte differentiation in mosquito [14], and for analyzing hemocyte-mediated phagocytosis in *Apis mellifera* [15].

Cytokines are highly evolutionarily conserved [16]; however, few studies have confirmed their presence in insects. Eleftherianos et al. collected data on cytokines in Drosophila, some of which are homologues of vertebrate cytokines: Eiger (eda-like cell death trigger) a homolog of the mammalian TNF-α, Spätzle (Spz) a homolog of the mammalian IL-17F, Unpaired (Upd) (Upd1, Upd2, Upd3) a homolog of mammalian type I cytokines and vertebrate leptin-like (interleukin 6 (IL-6) family) [17]. A sequence homologous to an interferon consensus response element has been reported in the diptericin promoter of *Drosophila* [18], and the lepidopteran cytotoxic molecule Gallysin 2, a potential analogue of TNF- family, has been isolated from *G*. *mellonella* [19]. Non-activated granular cells from *G*. *mellonella* or *Estigmene acraea* larvae hemocytes have shown strong positive reactions to polyclonal antibodies (pAb) against IL-1α and TNF-α, with *G*. *mellonella* plasmatocytes appearing to be less positive [20]. In addition, TNF-like molecules have been observed in the plasmatocytes and granular cells of *Calliphora vomitoria* haemocytes [21]. However, no methods currently exist for intracellular cytokine detection in insects based on flow cytometry; as such, there is a great need to develop a protocol using hemocytes.

The aim of the experiment was to develop a reproducible and efficient protocol for ICCS with *G*. *mellonella* hemocytes. The presented protocol optimizes the following parameters: buffer compositions, cell centrifugation conditions, fixation and permeabilization conditions, and antibody incubation time.

## Materials and methods

The protocol described in this peer-reviewed article is published on protocols.io dx.doi.org/10.17504/protocols.io.b3xxqppn and is included for printing as S1 File with this article.

## Expected results

Due to the morphological variety of the *G*. *mellonella* hemocyte subpopulations, their strong adhesion potential and ability to form networks (S1 Fig), it is not possible to apply the available ICCS procedures developed for mammalian cells. Therefore, it was necessary to develop a new protocol to obtain reproducible results.

Fluorescence microscopy studies revealed the presence of a protein similar to human IFN gamma in the culture of *G*. *mellonella* hemocytes (S2 Fig). The protein was immunolocalized using IFN gamma monoclonal antibodies from two different producers: Invitrogen (a part of Thermo Fisher Scientific) and from Enzo Life Sciences. Goat anti-Rabbit IgG (H+L) Secondary Antibody, DyLight 488 (Invitrogen) was used as a secondary antibody. The presence of IFN gamma was confirmed by intense fluorescence on the FITC channel (green). It was therefore decided to use these antibodies in the development of the ICCS protocol, and the cytometry results were expected to reveal a high percentage of positive cells.

The presence of IFN gamma- like protein in *G*. *mellonella* hemocytes was also confirmed using two-dimensional gel electrophoresis (2-DE) proteomic analysis. Total protein extraction was performed using CytoBuster Protein Extraction Reagent (Merck) with the addition of 1:100 Protease Inhibitor Cocktail Set III (Merck). Protein samples were prepared using ReadyPrep 2-D cleanup kit (Bio Rad). Approximately 90 μg of protein was loaded onto an immobilized pH gradient strip holder containing 7-cm strips with linear gradients ranging from pH 3 to 7 (Bio Rad). The strips were hydrated for 16h at 20°C, and then placed into a PROTEAN i12™ IEF system (Bio Rad). IEF was performed as follows: 250V for 15 min, 2500V for 2h and a 2500V gradient for 10000 V·h. After focusing, the strips were equilibrated. The proteins were separated in the second dimension using 12% SDS polyacrylamide gels on an Mini-PROTEAN Tetra cell (Bio Rad). The gels were stained using Pierce Silver Stain Kit (Thermo Scientific). LC-MS-MS/MS protein identification was performed by Mass Spectrometry Laboratory, Institute of Biochemistry and Biophysics, Polish Academy of Sciences, Warsaw, Poland. The protein was detected by Western Blot, using the same primary antibody as for fluorescence microscopy and for the development of the flow cytometry protocol. Goat anti-Rabbit IgG (H+L) Secondary Antibody, HRP (Invitrogen) was used as a secondary antibody. Proteomic analysis showed the presence of IFN gamma- like protein in the hemolymph of *G*. *mellonella* larvae. NCBI Blast search confirmed 33% protein sequence coverage to IFN gamma sequence from *Homo sapiens*. The proteomic results are shown in S3 Fig. For comparison human IFN gamma shows about 40% sequence homology with mouse IFN gamma. On the other hand chicken IFN gamma (ChIFN-gamma) has 30–35% homology with human IFN gamma [22]. Positive identification of IFN gamma using fluorescence microscopy and proteomic methods became the basis for the development of the ICCS protocol.

The flow cytometry protocol employed hemocytes collected from last instar (7^th^) *G*. *mellonella* larvae: the hemolymph collection method is given in S1 Video. However, it should be remembered that last stage larvae cease feeding on the fifth day, i.e. 2–3 days before pupation, and this should be taken into account in experiments where test substance/substances are administered with food.

Due to the ability of plasmatocytes and granulocytes to network and adhere, the first step was to develop a buffer with the addition of anticoagulants. The most frequently-used buffer in studies with insect cells is IPS (150 mM NaCl, 5 mM KCl, 10 mM tris HCl, pH 6.9). However, no differences were observed in the flow cytometry results when using IPS and PBS, and in our opinion, using PBS may be more efficient and faster, e.g. due to the possibility of using ready-made commercially-available tablets for buffer preparation. Other components that have been used to prevent coagulation of *G*. *mellonella* hemocytes include EDTA, sodium citrate and citric acid in various proportions [23–26]. In our protocol, we indicate 10mM EDTA and 30mM sodium citrate as the optimal anti-coagulant supplement. Our addition of 0.1mM PTU is also very important in order to prevent melanization.

**Fig 1:**
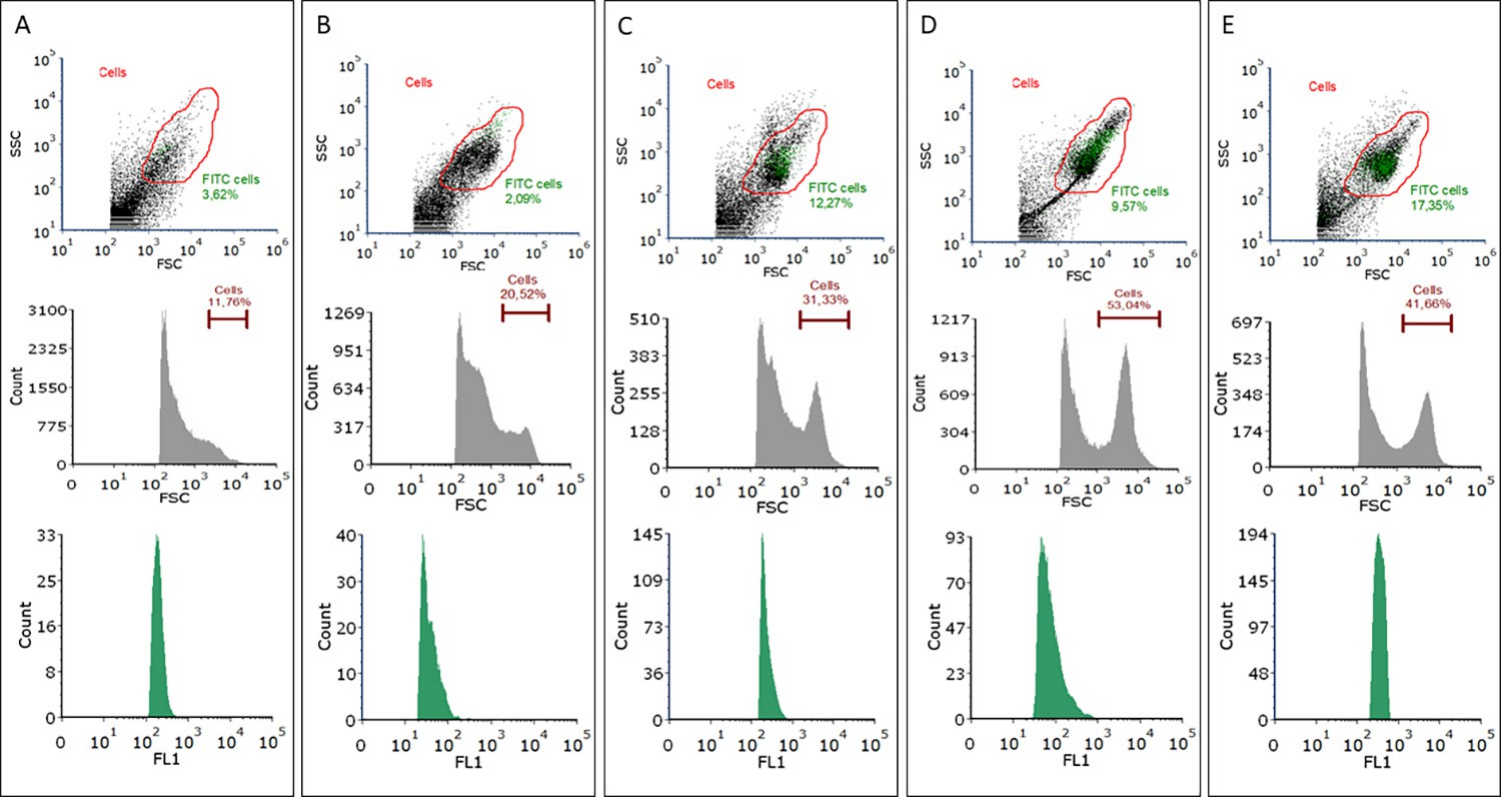
The development of the ICCS protocol for intracellular cytokine (IFN gamma) staining based on flow cytometry in *Galleria mellonella* hemocytes. [A, B] selection of the centrifugation conditions: A- 10 min 400 x g, B- 10 min 300 x g; [C] PFA concentration: 2% appeared too low; [D, E] incubation time with primary antibody: D- 90 min, E- 8h. The results are presented as follows: FSC vs SSC, red gate- haemocytes (dot plot); Count vs FCS (histogram); Count vs FL1 extracted from red gate (histogram).

Other important elements in the development of the protocol were the selection of the centrifugation conditions used for the preparation of the hemolymph samples, and the method of washing between the individual stages. Indeed, very different recommendations regarding centrifugation speed (200–800 x g) and time (3–15 minutes) are given regarding the development of ICCS using mammalian blood cells [1, 27]. The *G*. *mellonella* hemocytes are more delicate than mammalian cells, and excessive speed and centrifugation times will result in their breakdown. To illustrate, a dot plot (FSC vs SSC) and histograms (count vs FCS and count vs FL1) from the cytometric analysis of hemocytes treated with 400 x g centrifugation for 10 minutes are given in Fig 1A. No clear cloud of cells can be seen in the gate marked in red, and a large number of disintegrated fragments are present. After series of experiments we concluded that the optimal centrifugation condition for hemocytes is 300 x g for 5 minutes, and these were applied for all steps of the protocol.

The greatest obstacles to efficient intracellular cytokine detection are the problems associated with cell fixation and permeabilization, and their combination with cell staining. For cell fixation, 1–4% PFA is most often recommended. Some authors have also recommended the use of 4% PFA in PBS when using insect hemocytes, particularly for microscopy [28, 29]. In our protocol, we also found this PFA concentration to be the optimum for the fixation of *G*. *mellonella* hemocytes. At a lower concentration, i.e. 2% PFA, insufficient cell fixation was observed; the cytometric analysis also revealed hemocyte disintegration and insufficient penetration of the antibody (Fig 1C). In turn, the most frequently-recommended permeabilization buffer for working with insect hemocytes is 0.1–0.5% Triton X-100 [30–32]. We decided in our protocol to use the lowest recommended concentration (0.1%) as this allowed for good permeabilization of the cell membrane and effective penetration of the antibody into the cytoplasm.

The last step to be refined was staining with primary and secondary antibodies. In order to confirm the universality and repeatability of the protocol, anti-IFN gamma monoclonal antibodies [species reactivity: human, host: rabbit] from two different producers were used: from Invitrogen (a part of Thermo Fisher Scientific) and from Enzo Life Sciences. Both antibodies were used at the manufacturer’s recommended 1: 100 working dilution, which also turned out to be optimal in our experiments. The ICCS protocols developed for mammalian cells recommend incubation times of 30 to 90 minutes with the primary antibody. In the present study, after a 90-minute incubation, the percentage of cells showing fluorescence on the FL1 channel was less than 10% (Fig 1D). Therefore, the time was increased for subsequent samples, and even incubation for eight hours did not give satisfactory results (Fig 1E). Only overnight incubation at 4°C allowed the expected positive results to exceed 90%. Goat anti-Rabbit IgG (H+L) Secondary Antibody, DyLight 488 (Invitrogen) was used as a secondary antibody at a dilution of 1:100. The incubation protocol recommended elsewhere, i.e. 90 minutes at room temperature, also turned out to be optimal in our experiment.

**Fig 2:**
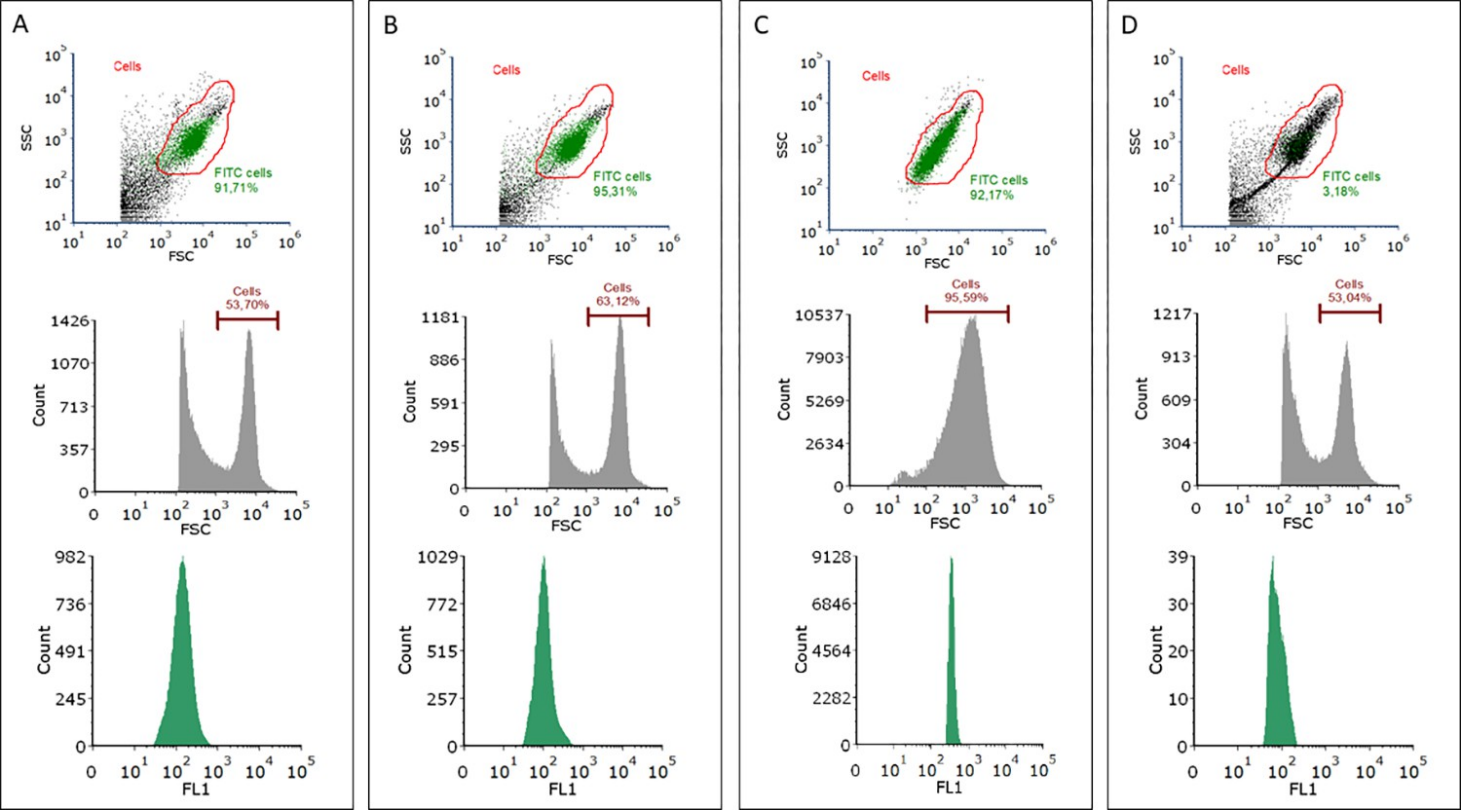
Intracellular cytokine (IFN gamma) staining based on flow cytometry in *Galleria mellonella* hemocytes using the developed ICCS protocol. A- staining with primary antibody purchased from Invitrogen; B- staining with primary antibody purchased from Enzo Life Sciences; C- positive control using eBioscience Mouse Cytokine Positive Control Cells (Invitrogen); D- autofluorescence control. The results are presented as: FSC vs SSC, red gate- hemocytes (dot plot); Count vs FCS (histogram); Count vs FL1 extracted from red gate (histogram).

The results of the IFN gamma flow cytometry analysis in *G*. *mellonella* hemocytes performed according to the developed protocol are given as dot plots and histograms in Fig 2A and 2B. The SSC vs FSC dot plots reveal a clear population of hemocytes (gate red) and the count vs FSC histograms indicate a strong peak from the cells. It is clearly visible that the cells are not broken down and form a compact population. A high percentage of positive cells fluorescing on the green channel (FL1) was recorded.

eBioscience Mouse Cytokine Positive Control Cells [Invitrogen] were used as positive controls (Fig 2C); these cells were previously fixed by the manufacturer using PFA. The next steps of the IFN gamma detection procedure were carried out in accordance with our developed protocol. A high percentage of positive cells was also found. The results obtained from the hemocyte autofluorescence controls, i.e. unstained cells treated exactly the same way as the tested cells, are illustrated in Fig 2D. The developed protocol does not cause excessive autofluorescence of *G*. *mellonella* hemocytes. All flow cytometry analysis were performed using CyFlow Cube 8 (Sysmex), and the data was analyzed with FCS Express 7 Research Edition (DeNovo Software).

**Fig 3:**
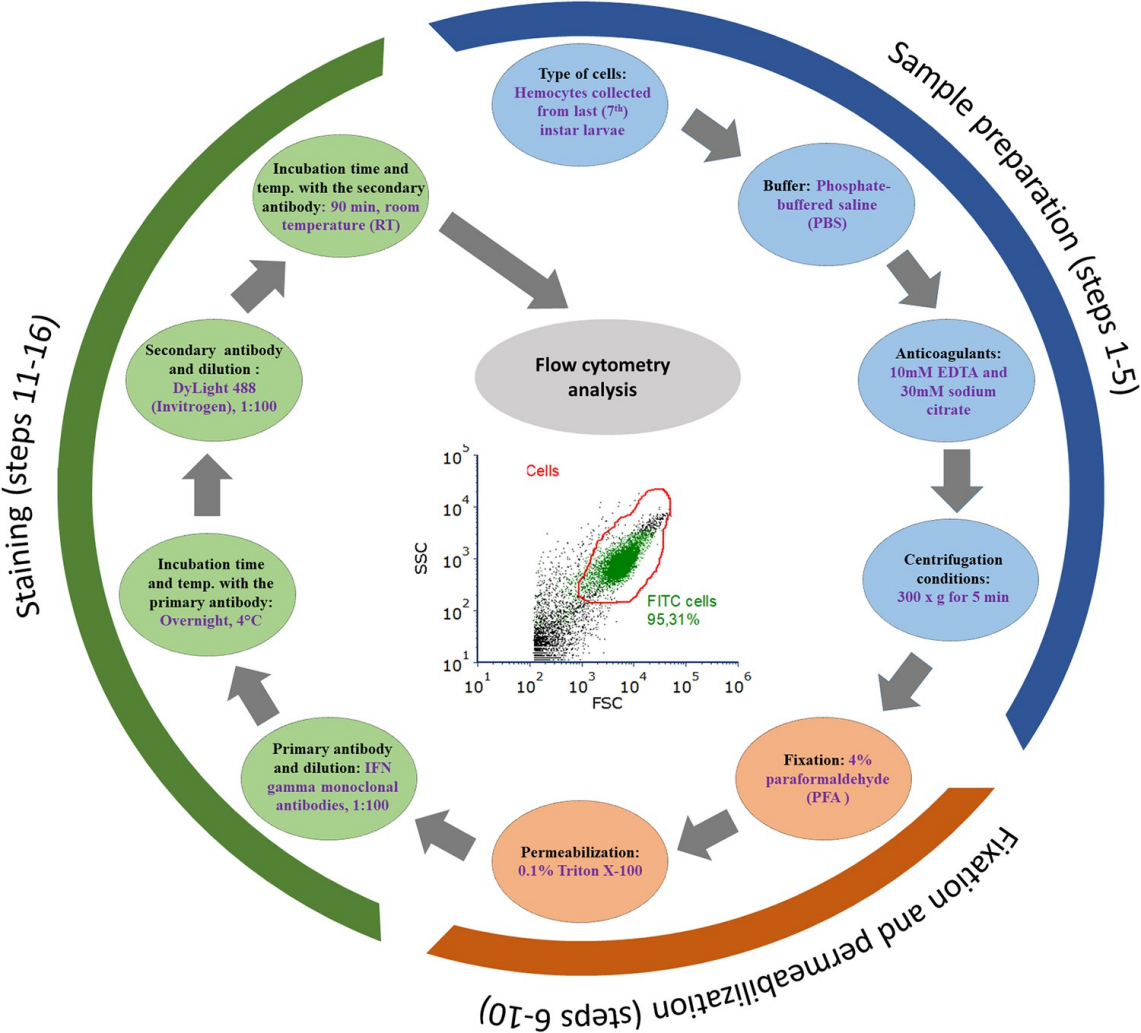
Optimized stages for flow cytometry staining for intracellular cytokine IFN gamma in *Galleria mellonella* hemocytes.

The steps of the developed protocol are illustrated in Fig 3.

The protocol can be used for intracellular cytokine detection in *G*. *mellonella* hemocytes, and as a suggestion for optimizing the fixation, permeabilization and cell staining procedure for other insect cells.

## Supporting information

S1 File Step-by-step protocol, also available on protocols.io. (PDF)

S1 Fig In vitro culture of hemocytes obtained from *Galleria mellonella* larvae. A- 24 hour cell culture; individual cell subpopulations: PR- prohemocyte, PL- plasmatocyte, GR- granulocyte, SF- spherulocyte, OE- oenocytoid.
(TIF)

S2 Fig Immunofluorescence localization of IFN gamma in *Galleria mellonella* hemocytes. A- staining with primary antibody purchased from Invitrogen; B- staining with primary antibody purchased from Enzo Life Sciences; β- actin [orange] was stained by ActinRed 555 ReadyProbes Reagent [Invitrogen]; cell nuclei [blue] were stained with Hoechst [Enzo Life Sciences]; Goat anti-Rabbit IgG [H+L] Secondary Antibody, DyLight 488 [Invitrogen] was used as a secondary antibody.
(TIF)

S3 Fig Separation by two-dimensional [2DE] gel electrophoresis [IEF + SDS-PAGE] and immunodetection of IFN gamma-like protein from *Galleria mellonella* hemocytes for peptide sequencing. A- 2-DE gel stained using Pierce Silver Stain Kit [Thermo Scientific], B- Western Blot membrane, C- *Homo sapiens* IFN-gamma amino acid sequence, peptides matching the sequence of the protein are marked in bold red.
(TIF)

S1 Video Hemolymph collection procedure from *Galleria mellonella* larvae. (RAR)
