# Supplementary material for: Intracellular cytokine detection based on flow cytometry in hemocytes from Galleria mellonella larvae: A new protocol
Source: PLoS One. 2022 Sep 29;17(9):e0274120. doi: 10.1371/journal.pone.0274120 (PMC9521830; doi:10.1371/journal.pone.0274120)
Supplement: S1 File — (PDF) [file pone.0274120.s001.pdf]

Intracellular cytokine detection based on flow cytometry in hemocytes from *Galleria mellonella* larvae

Anna Katarzyna Wrońska[1], Agata Kaczmarek[1], Mieczysława Irena Boguś[1]

[1]Witold Stefański Institute of Parasitology, Polish Academy of Sciences, Warsaw, Poland

dx.doi.org/10.17504/protocols.io.b3xxqppn

Anna Katarzyna Wrońska
Witold Stefański Institute of Parasitology, Polish Academy o...

Invertebrates are becoming increasingly popular models for research on the immune system. The innate immunity possessed by insects shows both structural and functional similarity to the resistance displayed by mammals, and many processes occurring in insect hemocytes are similar to those that occur in mammals. However, the use of insects as research models requires the development of methods for working with hemocytes. Therefore, a protocol for intracellular cytokine detection in *Galleria mellonella* larvae hemocytes based on flow cytometry has been developed. It describes the anticoagulant composition of the buffer, the optimal conditions for hemocyte permeabilization and fixation, as well as the conditions of cell centrifugation to prevent cell disintegration. A key element is the selection of staining conditions, especially the length of the incubation time with the primary antibody, which turned out to be much longer than recommended for mammalian cells. The development of these individual steps allowed for the creation of a reproducible protocol for cytokine detection using flow cytometry in wax moth hemocytes. This will certainly facilitate the development of further protocols allowing for wider use of insect cells in immunological research.

DOI

dx.doi.org/10.17504/protocols.io.b3xxqppn

Anna Katarzyna Wrońska, Agata Kaczmarek, Mieczysława Irena Boguś . Intracellular cytokine detection based on flow cytometry in hemocytes from Galleria mellonella larvae. **protocols.io**
https://dx.doi.org/10.17504/protocols.io.b3xxqppn

National Science Centre, Poland
Grant ID: 2019/35/D/NZ6/01685

cytokine detection , flow cytometry, Galleria mellonella, hemocytes

protocol ,

Jan 17, 2022

Jan 19, 2022

57047

PBS+AB: PBS (137 mM NaCl, 2.7 mM KCl, 8 mM Na_2HPO_4, and 2 mM KH_2PO_4) with 0.1 mM PTU (phenylthiourea) and anticoagulant buffer (AB): 10mM EDTA (ethylenediaminetetraacetic acid), 30mM sodium citrate

Fixation buffer: 4% paraformaldehyde in PBS+AB.

Permeabilization buffer: 0.1% Triton X-100 in PBS+AB.

Paraformaldehyde is:

- flammable solid
- carcinogen (IARC Group 1 – confirmed human carcinogen)
- sensitizer (skin and pulmonary)
- irritant (skin, eye, and respiratory tract.
- reproductive toxin (as a result of the formation of formaldehyde which is a decomposition product of paraformaldehyde)
- toxic (by skin contact and inhalation)

Breathing paraformaldehyde powders will irritate the nose and throat after prolonged exposure causing a cough, shortness of breath and possible lung damage.

Sample preparation 40m

1 Before bleeding, wash the larvae with distilled water (15 seconds) and then immerse briefly (5 seconds) in 70% (v/v) ethanol to sterilize their surfaces, thus reducing the chance of contamination of hemolymph samples. 2m

2 Collect the hemolymph into sterile tube from the larvae through the incision made in the last proleg 10m

3 100 µl of fresh hemolymph collected from ten larvae suspend in 100 µl of PBS+AB buffer 3m

4 Centrifuge cells **300 x g, 4°C, 00:05:00** 5m

5 Carefully remove the supernatant and resuspend the cell pellet in 100 µl of PBS+AB buffer. Repeat this step three times. 20m

Fixation 35m

6 Carefully remove the supernatant and resuspend the cell pellet in 500 µl of fixation buffer. 10m
Incubate for 00:10:00 Room temperature

7 Centrifuge at 300 x g, 4°C, 00:05:00 and remove the fixation buffer. 5m

8 Wash fixed cells with PBS+AB buffer. Centrifuge at 300 x g, 4°C, 00:05:00 and discard the supernatant. Repeat this step three times. 20m

Permeabilization 30m

9 Resuspend the cell pellet in 500 µl of permeabilization buffer with 0.5% BSA (bovine serum albumin). Incubate for 00:10:00 Room temperature 10m

10 Wash the fixed/permeabilized cells with permeabilization buffer. Centrifuge at 300 x g, 4°C, 00:05:00 and discard the supernatant. Repeat this step three times. 20m

Staining 18h 20m

11 Dilute the primary antibody with permeabilization buffer to an optimal working concentration and resuspend the fixed/permeabilized cells with primary antibody solution. Incubate Overnight 4 °C in the dark. 16h

12 Centrifuge at 300 x g, 4°C, 00:05:00 and remove the supernatant. 5m

13 Wash with permeabilization buffer and centrifuge at 300 x g, 4°C, 00:05:00 and discard the supernatant. Repeat this step three times. 20m

14 Dilute the fluorescent-conjugated secondary antibody with permeabilization buffer for an optimal working concentration and resuspend the cell pellet with secondary antibody solution. Incubate for 01:30:00 Room temperature in the dark. 1h 30m

15 Wash with permeabilization buffer and centrifuge at 300 x g, 4°C, 00:05:00 and discard the supernatant. Repeat this step three times. 20m

16 Resuspend cells in 1000 µl PBS+AB buffer. Before cytometric analysis, filter the resuspended 5m

cells using cell strainers, mesh size: 30µm.
