# Supplementary figures and images for: Intracellular cytokine detection based on flow cytometry in hemocytes from Galleria mellonella larvae: A new protocol
Source: PLoS One. 2022 Sep 29;17(9):e0274120. doi: 10.1371/journal.pone.0274120 (PMC9521830; doi:10.1371/journal.pone.0274120)

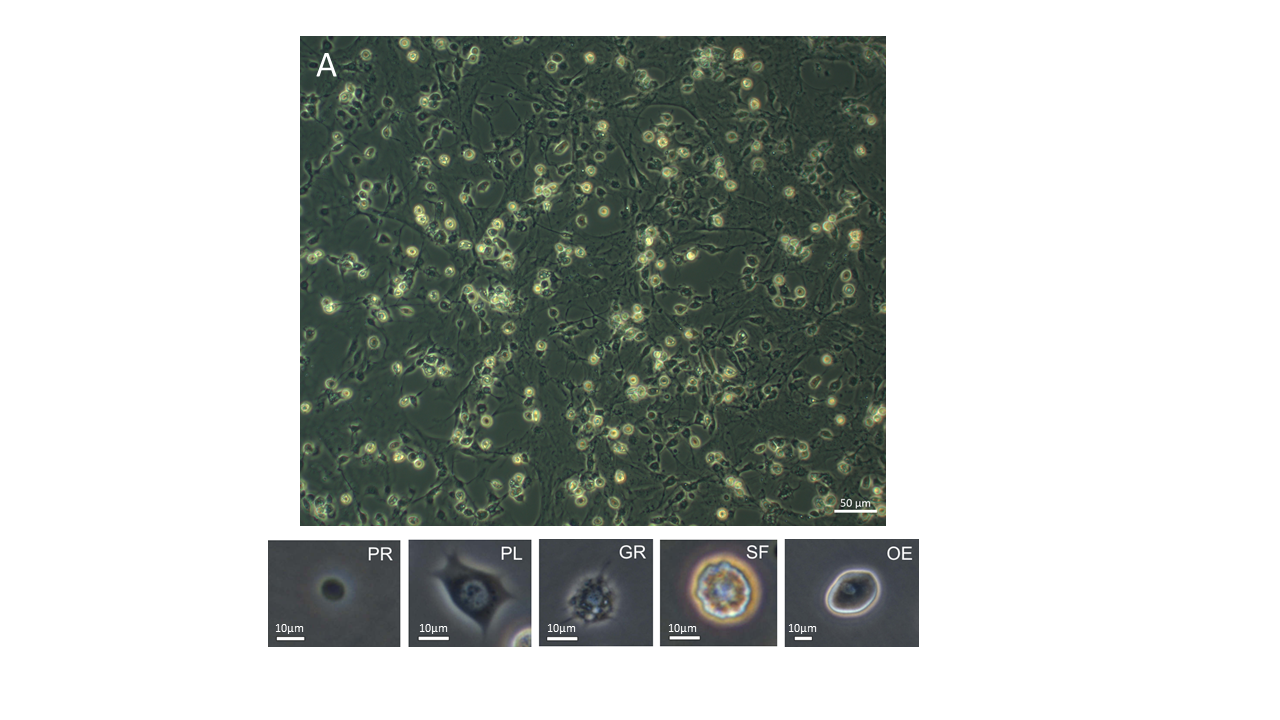

Supplement: S1 Fig — A- 24 hour cell culture; individual cell subpopulations: PR- prohemocyte, PL- plasmatocyte, GR- granulocyte, SF- spherulocyte, OE- oenocytoid. (TIF) [file pone.0274120.s002.tif]

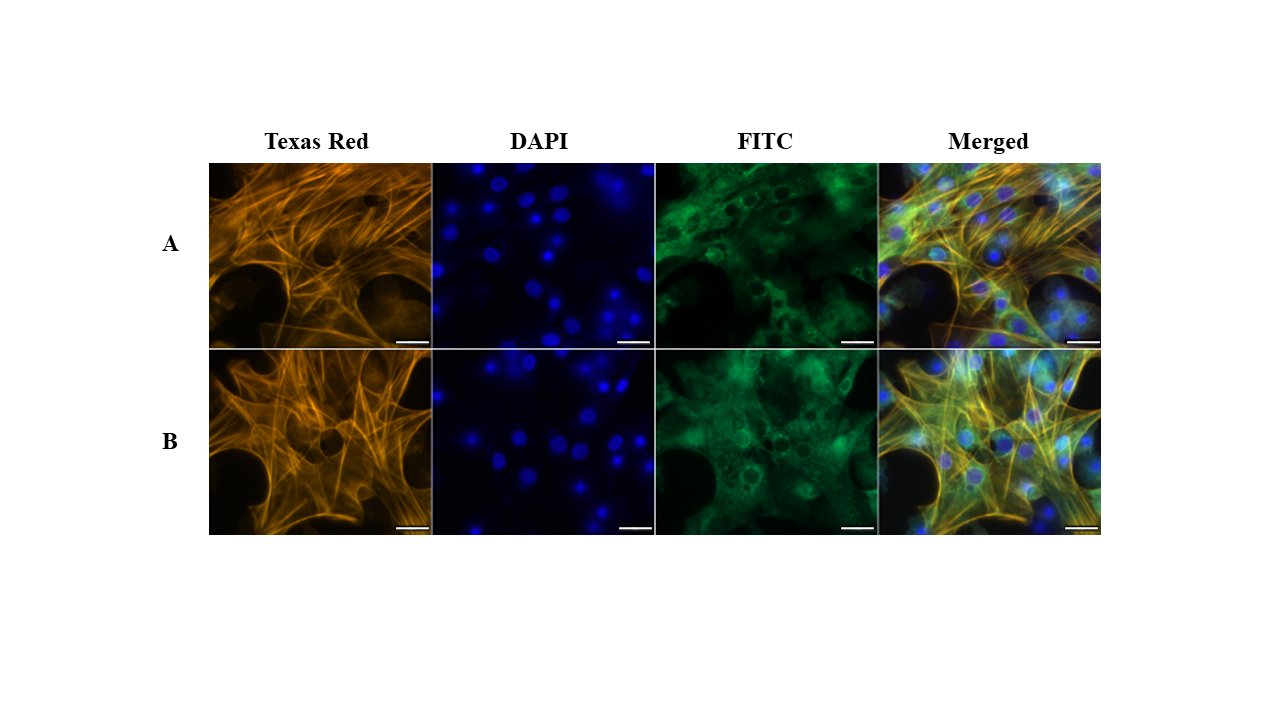

Supplement: S2 Fig — A- staining with primary antibody purchased from Invitrogen; B- staining with primary antibody purchased from Enzo Life Sciences; β- actin [orange] was stained by ActinRed 555 ReadyProbes Reagent [Invitrogen]; cell nuclei [blue] were stained with Hoechst [Enzo Life Sciences]; Goat anti-Rabbit IgG [H+L] Secondary Antibody, DyLight 488 [Invitrogen] was used as a secondary antibody. (TIF) [file pone.0274120.s003.tif]

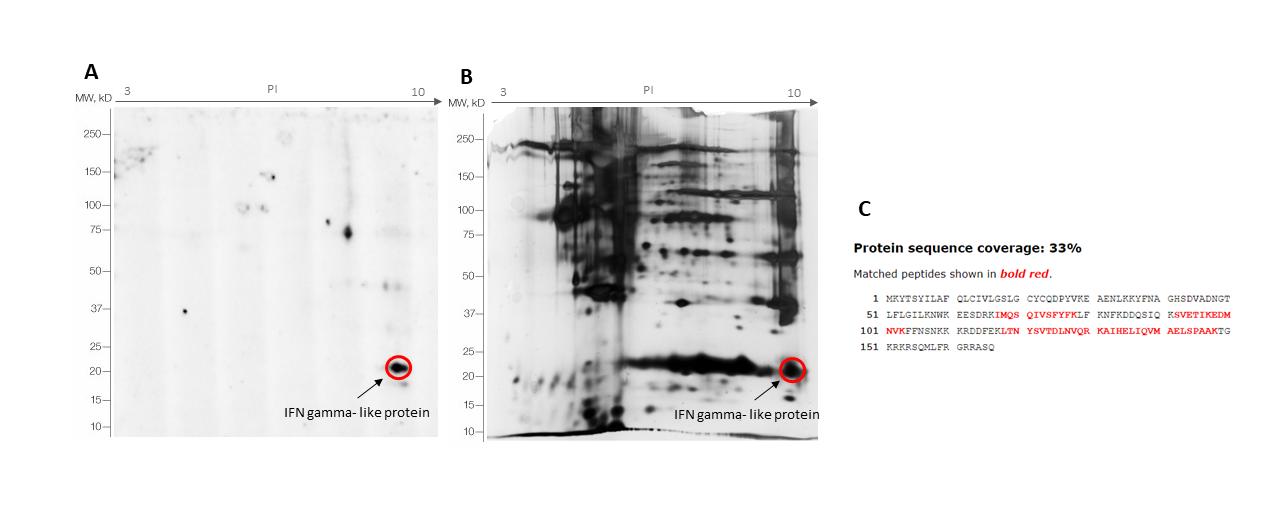

Supplement: S3 Fig — A- 2-DE gel stained using Pierce Silver Stain Kit [Thermo Scientific], B- Western Blot membrane, C- Homo sapiens IFN-gamma amino acid sequence, peptides matching the sequence of the protein are marked in bold red. (TIF) [file pone.0274120.s004.tif]
